# Impact of insurance type on outpatient mental health treatment of US adults

**DOI:** 10.1371/journal.pmen.0000299

**Published:** 2025-05-09

**Authors:** Lydia A. Chwastiak, Scott Graupensperger, Heather Ringeisen, Mark Edlund, Heidi Guyer, Natalie Bareis, Maria Monroe-Devita, Lisa Dixon, Scott Stroup, Jeffrey Swanson, Marvin Swartz, Elizabeth Sinclair Hancq, Robert Gibbons, Ronald C. Kessler, Mark Olfson

**Affiliations:** 1 Department of Psychiatry and Behavioral Sciences, University of Washington School of Medicine, Seattle, Washington, United States of America; 2 RTI International, Research Triangle Park, North Carolina, United States of America; 3 Department of Psychiatry, Columbia University School of Medicine, New York State Psychiatric Institute, New York, New York, United States of America; 4 Department of Psychiatry and Behavioral Sciences, Duke University School of Medicine, Durham, North Carolina, United States of America; 5 Treatment Advocacy Center, Arlington, Virginia, United States of America; 6 Department of Public Health Sciences, University of Chicago, Chicago, Illinois, United States of America; 7 Department of Health Care Policy, Harvard Medical School, Boston, Massachusetts, United States of America; University of Milano–Bicocca: Universita degli Studi di Milano-Bicocca, ITALY

## Abstract

The mental health treatment gap in the US continues to be a major public health challenge. Even individuals with health insurance face substantial barriers to care, including high costs, insufficient coverage and inaccurate provider directories. Policies to address the treatment gap require updated population-based information about whether treatment rates vary by type of insurance. The current study aimed to compare past-year outpatient mental health treatment across insurance types (private, Medicare, Medicaid, other, or none), in the household sample of non-elderly adults in the Mental and Substance Use Disorder Prevalence Study (MDPS), (n = 4,640). MDPS, fielded October 2020 through October 2022, identified 12-month prevalence of mental disorders and rates of treatment among US adults from interviews by trained clinicians using the Structured Clinical Interview for DSM-5. Logistic regressions estimated odds of treatment among participants with a past-year MDPS diagnosis across insurance types, after adjusting for age, sex, race/ethnicity, income level, diagnosis, and functional impairment. Analyses were weighted to reflect the US adult population. 60.2% of the 1,833 participants with an MDPS mental disorder received outpatient treatment in the past year. Compared to participants with private insurance, those with no insurance had lower odds of outpatient treatment (AOR = 0.37 [0.16-0.87]). Participants with Medicare had higher odds of treatment (AOR = 4.25 [1.56-11.64]), suggesting that individuals with complex and disabling illness were least likely to have treatment disruptions during the early phases of the pandemic. Differences between groups decreased as the pandemic progressed, but utilization of services only significantly increased among individuals with private insurance. Persisting mental health treatment gaps in the US vary by type of health insurance, which warrants extensive policy reforms.

## 1. Introduction

The substantial fraction of people in the US with mental disorders who do not receive treatment (“the mental health treatment gap”) has been recognized as a major public health challenge for more than twenty years [1]. In the 2019 National Survey on Drug Use and Health (NSDUH), 39% of adults with a mental disorder did not receive any outpatient or prescription treatment in the previous 12 months [2]. While attitudinal barriers (e.g., perceived stigma or beliefs that treatments are ineffective) contribute to low rates of mental health treatment [3–5], substantial structural barriers to care drive the mental health treatment gap. These include high costs of care, lack of insurance or insufficient coverage for services, and not knowing where to go for care or being able to get an appointment [6,7]. Analyses of 2019 NSDUH data revealed that 23% of US adults with moderate-severe anxiety or depression who were not receiving treatment had skipped or delayed therapy due to cost [2].

According to US Census Bureau data, in 2023, 92% of US adults had health insurance for all or part of the year; 65.4% of US adults had private health insurance, the vast majority of which is employment-based; and 36.3% had some form of public health insurance [8]. Previous studies suggest that among people with mental disorders, those who lack health insurance are the least likely to receive treatment [9–11], but individuals who have health insurance also face structural barriers to care, including insufficient coverage for services or not being able to get an appointment [6,7,12]. Because commercial health plans pay substantially less for in-network mental health services than for services provided by other specialists [13], psychiatrists are less likely than other medical specialists to participate in private insurance networks, with one-third of psychiatrists reporting they do not accept new patients with private insurance [14]. Mental health services are up to six times more likely than general medical services to be delivered by an out-of-network provider [15].

Individuals who have public insurance (primarily Medicare and Medicaid) may face additional barriers to mental health care. Medicare is a federal program for all people older than 65 and for people younger than 65 who have disabilities, while Medicaid is a joint state and federal program for people with low incomes. Medicare is a major source of coverage for non-elderly individuals with a disabling mental illness, but enrollees must negotiate inaccurate provider directories and insurance denials, particularly for expensive treatments that have proliferated in recent years [16]. Similarly, high rates of “phantom providers” who are listed in directories but do not see Medicaid patients represent barrier to care for Medicaid enrollees [17]. Additional challenges for Medicaid enrollees include lower reimbursement rates than those of private insurance or Medicare: Medicaid fee-for-service payments for physician services may be nearly 30 percent below those of Medicare [17]. In 2022, Medicaid’s fee-for-service rates for commonly billed psychiatry services were 81% of those in Medicare [18].

Updated information on whether outpatient mental health treatment rates differ by type of insurance can inform policy decisions that address the current mental health treatment gap in the US. The current study used data from a national household sample of US adults aged 18–64 who met past-year criteria for one of six mental disorders (major depressive disorder, generalized anxiety disorder, posttraumatic stress disorder, schizophrenia spectrum disorder, bipolar disorder and obsessive-compulsive disorder) (N = 1,833) to compare outpatient mental health treatment rates across insurance types. Data were from the Mental and Substance Use Disorders Prevalence Study (MDPS), a large national epidemiologic study conducted between October 2020 and October 2022. As data were collected during the first two years of the COVID pandemic, the MDPS study provides a unique opportunity to evaluate whether differences in rates of mental health treatment across insurance types differed during different phases of the pandemic.

The current study aimed to 1) compare rates of any outpatient mental health treatment and number of visits among MDPS participants meeting criteria for one of six past-year mental disorder diagnoses across insurance types (private, Medicare, Medicaid, military or other, and none), and 2) evaluate whether differences in mental health treatment access across insurance types differed early in the pandemic (2020–2021) compared to later (2021–2022).

## 2. Methods

### 2.1. Participants

The MDPS was designed to identify prevalence estimates of seven mental disorders and rates of treatment in the US. Trained clinicians used the Structured Clinical Interview for DSM-5 (SCID-5) for past-year diagnosis of mental disorders [19]. The MDPS was the first national study to use DSM-5 based semi-structured clinical interviews within a large-scale population survey, addressing the major limitations of previous large psychiatric epidemiology studies that used only screening scales for anxiety and depression and typically did not include more serious mental disorders such as schizophrenia. The study was funded by the US Substance Abuse and Mental Health Services Administration (SAMHSA) and data collection occurred between October 1, 2020- September 30, 2022. The MDPS recruited participants from national samples of households and prisons and from convenience samples of psychiatric hospitals and homeless shelters. The current analyses include only the household sample, which was selected from an address-based sampling frame [20] via a stratified multi-stage area probability sampling methodology [21]. The MDPS weighting methodology accounted for differential probabilities of selection, nonresponse adjustments, and poststratification to align with U.S. Census demographic benchmarks (e.g., age, sex, race/ethnicity, marital status, and education level) [22]. A total of 25,572 households were included [17.4% weighted response rate (RR)]. The MDPS included a multi-stage household design to identify persons likely to have a mental disorder, particularly schizophrenia spectrum disorder, for enhanced data collection via a clinical interview. Within each household, up to two adults were randomly selected to participate in screening; 29,084 household participants completed a screening interview (67.4% weighted conditional RR). Completed screening interviews were used to classify the respondents into one of three hierarchical mental health risk strata: Stratum 1—those who reported experiencing psychotic symptoms or receiving disability payments because of schizophrenia, Stratum 2—those who reported experiencing symptoms of other MDPS disorders, and Stratum 3—those who reported no symptoms associated with these mental disorders. One hundred percent of respondents from Stratum 1 were invited to complete a clinical interview. The selection rates for the other two strata were adjusted during the study to meet clinical interview study objectives, including the analytic goal of obtaining a target number of participants. Specifically, over the course of study data collection the sampling rate for Stratum 2 was increased from 20% to 80% and the sampling rate for Stratum 3 was increased from 8% to 20%. This multistage design resulted in an enriched MDPS household clinical interview sample comprising more cases meeting criteria for mental disorders, particularly schizophrenia, than would have been achieved without screening*.* Clinical interviews were completed with 4,764 household participants (31.2% weighted conditional RR); household interviews were conducted either virtually using the Zoom^®^ platform or by telephone. The overall weighted household clinical interview response rate, accounting for the prior stages, was 3.7%. Additional details on MDPS background and methods has been provided elsewhere [22]. The current analyses were limited to a subsample of respondents who were non-elderly adults (18–64 years) who met past-year criteria for one of the MDPS mental disorder diagnoses, n = 1,833).

**Ethics statement:** The study was approved by the Advarra Institutional Review Board (protocol FG00030/ 021786). Individual written informed consent was obtained from all participants.

### 2.2. Health insurance

Type of health insurance was categorized into five mutually exclusive hierarchically ordered categories for the primary analyses. These categories were ordered according to which insurance type is the primary payer when individuals have multiple insurance types: private insurance, Medicare, Medicaid, other insurance, and no insurance. Participants who had more than one type of insurance were included in the first category that they met. For example, participants who had both private insurance and Medicare were included in the private insurance group, and those with Medicare and Medicaid were included in the Medicare group.

### 2.3. Clinical characteristics

#### 2.3.1. *Diagnostic assessment of mental disorders.*

Trained clinicians used the Structured Clinical Interview for DSM-5 (SCID-5) [19] for past-year diagnosis of seven mental disorders: major depressive disorder, generalized anxiety disorder, bipolar disorder, schizophrenia spectrum disorders, post-traumatic stress disorder, obsessive compulsive disorder, and anorexia nervosa. The SCID-5 has very good to excellent reliability and validity [23–25]. The current analyses are limited to six disorders: analyses do not include anorexia nervosa because of its very low prevalence. Interviewers also used the SCID-5 to diagnose four substance use disorders (alcohol, cannabis, stimulants and opioids). Analyses included a covariate “any substance use disorder” which is defined as meeting past-year SCID-5 criteria for one or more of these disorders.

#### 2.3.2. *Global assessment of functioning.*

Clinical interviewers completed the Global Assessment of Functioning (GAF) at the end of each clinical interview to assess functional impairment and symptom severity [26]. This widely-used clinician assessment of functioning ranges from 0-100, with lower scores indicating more severe impairment. A threshold of 50 or lower has been validated and is commonly used to reflect severe impairment [26].

### 2.4. Sociodemographic characteristics

Sociodemographic characteristics included age, sex at birth, race, ethnicity, education level, household income, and state of residence at the time of the interview.

### 2.5. Date of the interview

The date of the interview was also recorded. Time in months since March 2020 was used in the analyses as an indicator of phase of the pandemic.

### 2.6. Outpatient mental health treatment in the past year

The interview included questions about the use of any medication or outpatient treatment for a mental disorder. Participants were asked whether they “ever received professional counseling, medication or other treatment to help with mental health, emotions, or behavior.” Those who reported any lifetime treatment were asked about receipt of treatment in the past year. Participants who reported any outpatient mental health treatment were then asked about the number of visits they had in the past year.

### 2.7. Statistical analyses.

Analyses were based on weighted data and were conducted using R software.

Because we were interested in the strength of the independent associations of insurance type with past year outpatient mental health treatment among adults with MDPS disorders, the primary analyses used logistic regression to estimate odds of past year outpatient mental health treatment across insurance types (private, Medicare, Medicaid, other insurance, or no insurance), with private insurance as the reference category. The model adjusted for age, sex at birth, race, ethnicity, education, and household income, any substance use disorder, functional impairment (GAF score ≤50), state of residence, and date of interview.

We then examined the number of outpatient visits in the past year among participants with an MDPS diagnosis who had at least one visit (i.e., a zero-truncated model). Given the count outcome with positive skew (i.e., overdispersion), we used generalized linear models with a quasipoisson log-link function that appropriately modifies the variance structure to account for overdispersion while maintaining the mean-variance relationship observed in a Poisson distribution.

To examine moderating effect of time, given the progression of the COVID-19 pandemic during the study period, we ran subsequent models including interaction terms (i.e., Insurance type × date of interview in months since March 2020). As recommended for nonlinear models, including binary and count outcomes, we estimated and plotted the marginal effects across each insurance category [27]. From these subsequent models, we only report the product-term parameters in the tables, given that main effect estimates are not intuitively interpreted once interactions are added [28].

## 3. Results

### 3.1. Weighted prevalence of MDPS disorders across insurance types

The MDPS household sample included (n = 4,640) adults who were aged 18–64. Twenty-five percent of these non-elderly adults (n = 1,833) met past-year criteria for one or more of the six MDPS mental health diagnosis (95% CI: 22.5—27.6). Regarding weighted percents of each insurance category, 54.2% percent of this sample had private insurance; 7.7% had Medicare, 23.6% had Medicaid; 7.3% had military or other insurance and 7.3% were uninsured. As shown in Table 1, the weighted prevalence of the MDPS diagnoses varied by insurance type. The prevalence estimate of schizophrenia spectrum disorder was highest among Medicaid enrollees, 11.9% (95% CI 6.4-19.7). The weighted prevalence of severe functional impairment (i.e., GAF score ≤50) also varied by insurance category: 42.8% (95% C.I: 28.0—58.5) for those with Medicare, 38.3% (95% C.I: 27.3—50.2) for those with Medicaid, 19.4% (95% C.I: 10.1—31.7) for those with other insurance, 12.8% (95% C.I: 9.4—16.8) for those with private insurance, and 12.4% (95% C.I: 6.0—21.6) for those with no insurance.

**Table 1:** Characteristics of non-elderly participants in MDPS household sample with an MDPS diagnosis (n = 1,833), by insurance category.

	Weighted Prevalence or Mean [95% C.I.]				
	Private Insurance 54.2% [48.5, 59.7]	Medicare 7.7% [5.4, 10.8]	Medicaid 23.6% [19.1,28.7]	Other 7.3% [4.8, 10.9]	No Insurance 7.3% [5.0, 10.5]
Mean Age	35.8 [33.7, 38.0]	45.3 [42.4, 48.1]*	36.7 [33.4, 40.0]	31.1 [25.6, 36.6]	34.5 [29.8, 39.1]
Female	60.5% [53.8, 67.0]	58.5% [41.7, 74.0]	63.9% [53.0, 73.9]	36.2% [20.3, 54.5]	58.7% [36.4, 78.8]
Race					
White	77.5% [71.9, 82.6]	66.2% [51.3, 79.1]	66.7% [55.2, 77.1]	64.3% [37.2, 86.3]	66.4% [48.8, 81.4]
Black	8.5% [5.2, 12.9]	24.7% [14.0, 38.1]*	20.4% [10.6, 33.2]	12.6% [4.5, 25.7]	17.2% [7.0, 32.4]
Asian	5.9% [3.2, 9.6]	2.5% [0.4, 7.8]	3.3% [1.1, 7.4]	0.0% [0.0, 0.0]*	2.1% [0.5, 5.6]
Other	8.1% [5.4, 11.4]	6.6% [2.4, 13.6]	9.6% [5.2, 15.6]	23.1% [3.9, 57.0]	14.3% [3.9, 32.5]
Hispanic Ethnicity	9.7% [6.6, 13.4]	5.0% [2.1, 9.5]	12.3% [7.7, 18.3]	22.1% [8.9, 41.0]	23.2% [11.7, 38.3]
Household Income					
<$20k	7.0% [4.3, 10.5]	54.1% [37.3, 70.2]*	59.0% [48.2, 69.2]*	9.3% [3.2, 19.4]	45.0% [27.7, 63.1]*
$20k-$50k	25.1% [17.4, 34.1]	31.9% [17.7, 48.9]	29.3% [21.2, 38.3]	44.7% [23.1, 67.7]	31.5% [19.0, 46.2]
$51k-$75k	14.9% [11.2, 19.2]	9.4% [3.5, 19.0]	6.5% [3.8, 10.1]*	20.5% [9.3, 36.0]	15.1% [4.4, 33.2]
>$75k	52.9% [44.5, 61.2]	4.6% [1.5, 10.2]*	5.3% [2.3, 10.1]*	25.6% [13.6, 40.8]	8.4% [3.8, 15.4]*
Education					
Less than High School	2.4% [0.9, 4.9]	13.5% [5.5, 25.6]*	12.9% [7.1, 20.6]*	19.7% [2.3, 55.0]	29.8% [12.8, 51.7]*
High School or GED	39.3% [32.0, 47.0]	60.3% [43.4, 75.6]	64.6% [54.4, 74.1]*	51.3% [29.5, 72.8]	58.5% [39.6, 75.9]
College or Associate Degree	44.9% [36.3, 53.7]	25.2% [12.9, 41.1]	21.0% [14.0, 29.2]*	25.8% [13.6, 41.3]	11.1% [5.7, 18.8]*
Graduate or Professional Deg.	13.4% [9.6, 17.9]	1.0% [0.3, 2.2]*	1.5% [0.9, 2.5]*	3.1% [1.4, 6.0]*	0.6% [0.1, 1.6]*
Marital status					
Married	47.2% [37.9, 56.5]	23.9% [10.8, 41.7]	21.3% [14.8, 28.9]*	29.7% [16.3, 46.1]	11.0% [5.1, 19.8]*
Previously Married	7.9% [5.7, 10.7]	42.8% [27.0, 59.7]*	23.5% [14.4, 34.6]*	8.5% [3.3, 17.1]	23.0% [11.4, 38.2]*
Never Married	44.9% [35.8, 54.3]	33.2% [22.2, 45.7]	55.2% [44.5, 65.6]	61.7% [43.2, 78.2]	66.0% [48.8, 80.8]
Past-year MDPS diagnoses					
Schizophrenia Spectrum Disorder	1.5% [0.6, 3.0]	10.8% [4.9, 19.4]*	11.9% [6.4, 19.7]*	5.0% [0.9, 14.3]	0.3% [0.0, 0.8]
Bipolar Disorder	4.1% [2.6, 6.1]	18.1% [5.6, 38.3]*	10.3% [3.0, 23.4]	0.9% [0.2, 2.7]	2.2% [0.7, 5.0]
MDD	67.8% [58.9, 75.9]	45.0% [30.2, 60.4]	59.4% [48.8, 69.4]	57.0% [34.1, 78.1]	62.3% [38.0, 82.9]
GAD	45.7% [37.8, 53.8]	31.6% [20.0, 44.9]	40.8% [30.3, 51.9]	31.1% [10.4, 59.2]	28.7% [14.4, 46.6]
PTSD	11.0% [8.0, 14.5]	31.8% [16.0, 51.0]*	20.0% [13.7, 27.5]*	27.3% [13.9, 44.4]*	21.4% [4.8, 49.8]
OCD	8.9% [6.3, 12.0]	6.2% [2.9, 11.2]	10.2% [5.1, 17.6]	24.5% [5.2, 56.3]	10.0% [4.5, 18.3]
Poor Functioning (GAF ≤ 50)	12.8% [9.4, 16.8]	42.8% [28.0, 58.5]*	38.3% [27.3, 50.2]*	19.4% [10.1, 31.7]	12.4% [6.0, 21.6]
Past-year Substance Use Disorder	21.9% [14.2, 31.1]	14.0% [6.7, 24.3]	25.6% [15.7, 37.6]	17.6% [9.3, 28.8]	20.7% [8.2, 38.7]

Comparisons on each demographic variable were made between private insurance as the reference category and the other insurance categories using weighted logistic regression (Gaussian for age comparisons). Given the large number of tests and inflated type-I error rate, we considered only *p* < .01 as significant, as identified with the star.

### 3.2. Outpatient mental health treatment in the past year across insurance types

**Fig 1:**
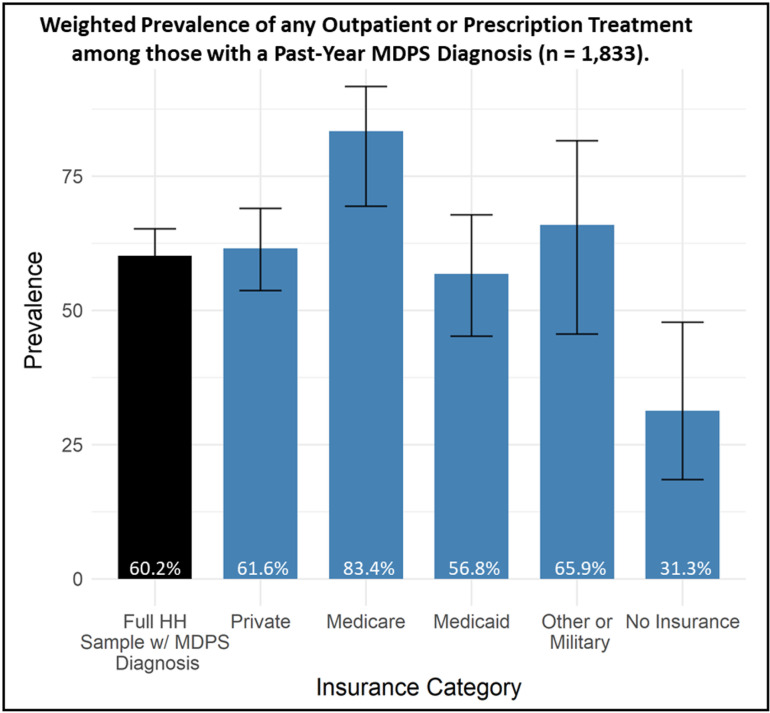
Weighted prevalence of any outpatient mental health visit or prescription medication among participants meeting past-year criteria for MDPS disorder (n = 1,833).

Among the 1,833 participants who met past-year criteria for any MDPS diagnosis, 60.2% (95% C.I: 55.2—65.2) received any outpatient mental health treatment in the past year. As shown in Fig 1, treatment was lowest among participants with no insurance (31.3%, 95% C.I: 17.8—47.4) and those with Medicaid (56.8%, 95% C.I: 45.2—67.9). Adjusted regressions revealed significant differences with respect to outpatient mental health treatment in the past year across insurance types (Table 2). Participants with no insurance (AOR = 0.37 [0.16, 0.89]) had significantly lower odds of any mental health treatment, compared to those with private insurance, while those with Medicare had significantly higher odds (AOR = 4.25 [1.59, 11.36]) of past-year treatment. In this model, participants who were Black or Asian American, or of Hispanic ethnicity, were also significantly less likely to receive any outpatient mental health treatment in the past year, relative to white participants. As participant age increased, the odds of treatment were slightly lower (conditional upon model covariates) indicating that past-year mental health treatment was slightly more likely among relatively younger adults, on average. Women were nearly twice as likely to have received any treatment, relative to men.

**Table 2:** Associations between insurance categories (relative to private insurance) and any outpatient or prescription mental health treatment in the past 12 months among those with any MDPS diagnosis (*N* = 1833).

	Adjusted Odds Ratio	95% CI	*P-*value
(Intercept)	2.59	[0.53, 12.67]	.232
Insurance Type			
Private Insurance (ref)			
Medicare	**4.25**	**[1.59, 11.36]**	**.005**
Medicaid	0.97	[0.52, 1.78]	.908
Other or Military	1.23	[0.60, 2.53]	.563
No Insurance	**0.37**	**[0.16, 0.89]**	**.027**
Age	**0.99**	**[0.97, 1.00]**	**.049**
Birth Sex (0 = Male, 1 = Female)	**1.94**	**[1.31, 2.88]**	**.002**
Race			
White (ref)			
Black	**0.30**	**[0.14, 0.64]**	**.003**
Asian	**0.30**	**[0.11, 0.83]**	**.022**
Other	1.05	[0.53, 2.10]	.881
Hispanic (0 = No, 1 = Yes)	**0.40**	**[0.24, 0.68]**	**.001**
Education			
Less than High School (ref)			
High School or GED	0.60	[0.24, 1.52]	.272
College or Associates Degree	1.00	[0.36, 2.74]	.996
Graduate or Professional Deg.	0.83	[0.29, 2.32]	.709
Household Income			
<$20k (ref)			
$20k–$50k	1.47	[0.80, 2.72]	.206
$51k–$75k	1.15	[0.58, 2.27]	.686
> $75k	1.08	[0.56, 2.06]	.818
Poor Functioning (GAF Score ≤ 50)	1.69	[0.92, 3.09]	.089
Past-Year Substance Use Disorder	1.02	[0.62, 1.68]	.950
Interview Date			
Time since March 2020 (in months)	1.00	[0.96, 1.04]	.914
**Interaction Effects from a Subsequent Model (Private Insurance is Referent)**			
Medicare × Interview Date	**0.85**	**[0.75, 0.96]**	**.010**
Medicaid × Interview Date	**0.89**	**[0.80, 0.99]**	**.038**
Other or Military × Interview Date	**0.84**	**[0.71, 0.99]**	**.039**
No Insurance × Interview Date	0.91	[0.78, 1.06]	.219

Coefficients in bold highlight *p* < .05. Model also controls for which state participants lived in.

**Fig 2:**
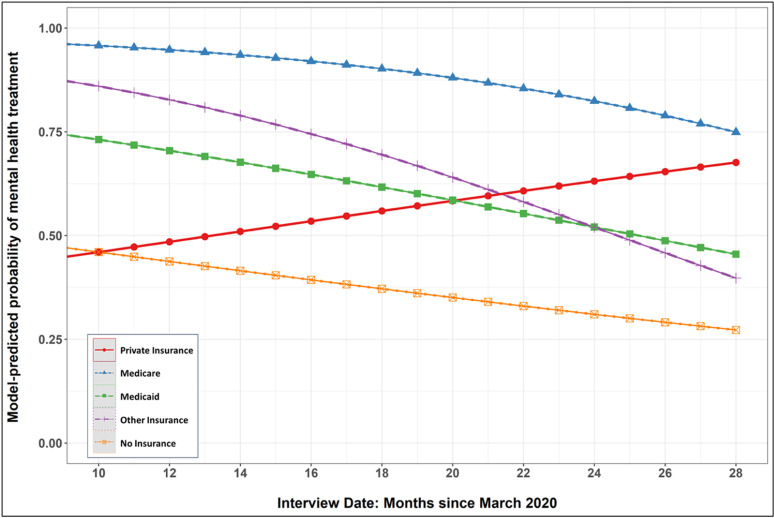
Probability of mental health treatment across insurance categories as pandemic progressed.

Adding the product-term interactions revealed that date of interview (i.e., months since March 2020) significantly moderated several of the insurance category comparisons. The effect differences between private insurance and Medicare, Medicaid, and Other were all reduced as the pandemic progressed. The marginal effects plotted in Fig 2 show that the model-predicted probability of mental health treatment increased with time for those with private insurance but decreased with time for those with all other insurance types.

**Fig 3:**
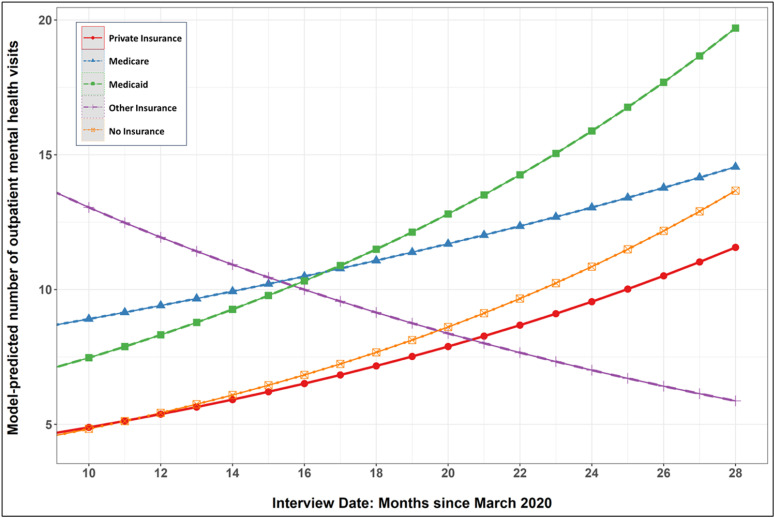
Mean number of mental health visits (among those with any visits) across insurance categories as pandemic progressed.

The final set of models estimated differences among insurance categories in terms of number of outpatient visits in the past year and was estimated among those who had at least one outpatient visit (Table 3). Relative to participants with private insurance, those with Medicaid had 63% more visits, on average (RR = 1.63, 95% C.I: 1.11—2.38). For reference, the weighted mean number of outpatient visits in the previous year (among those with at least one visit) was 30.7 for those with Medicaid, 20.8 for those with no insurance, 20.4 for those with other insurance, 19.6 for those with Medicare and 15.0 for those with private insurance. Adding the product-term interactions with date of interview identified only one instance of significant moderation: the difference in number of outpatient visits between private insurance and other insurance decreased with time. The marginal effects plot in Fig 3 shows that the number of outpatient visits increased, on average, as the date of interview progressed further away from March 2020 for all insurance types *other than* those with other insurance, which decreased over time.

**Table 3:** Associations between insurance categories (relative to private insurance) and number of outpatient mental health treatment visits in the past 12 months among those with any MDPS diagnosis and at least one mental health visit (*N* = 1185).

	Weighted Rate Ratio	95% CI	*P-*value
(Intercept)	**3.58**	**[1.30, 9.86]**	**.015**
Insurance Type			
Private Insurance (ref)			
Medicare	1.46	[0.92, 2.31]	.108
Medicaid	**1.63**	**[1.11, 2.38]**	**.013**
Other or Military	1.24	[0.83, 1.85]	.278
No Insurance	1.12	[0.57, 2.23]	.731
Age	**0.99**	**[0.98, 1.00]**	**.048**
Birth Sex (0 = Male, 1 = Female)	1.00	[0.75, 1.33]	.989
Race			
White (ref)			
Black	0.77	[0.51, 1.18]	.220
Asian	0.70	[0.36, 1.37]	.291
Other	1.33	[0.86, 2.06]	.195
Hispanic (0 = No, 1 = Yes)	0.80	[0.58, 1.11]	.177
Education			
Less than High School (ref)			
High School or GED	1.54	[0.89, 2.67]	.122
College or Associates Degree	1.74	[0.91, 3.31]	.093
Graduate or Professional Deg.	1.75	[0.97, 3.17]	.062
Household Income			
<$20k (ref)			
$20k-$50k	1.32	[0.91, 1.91]	.136
$51k-$75k	1.18	[0.73, 1.92]	.482
> $75k	1.13	[0.77, 1.64]	.531
Poor Functioning (GAF Score ≤ 50)	**1.45**	**[1.11, 1.90]**	**.009**
Past-Year Substance Use Disorder	**1.49**	**[1.00, 2.23]**	**.050**
Interview Date			
Time since March 2020 (in months)	**1.04**	**[1.01, 1.07]**	**.005**
**Interaction Effects from a Subsequent Model (Private Insurance is Referent)**			
Medicare × Interview Date	0.98	[0.91, 1.06]	.576
Medicaid × Interview Date	1.01	[0.94, 1.08]	.863
Other or Military × Interview Date	**0.91**	**[0.83, 1.00]**	**.046**
No Insurance × Interview Date	1.01	[0.90, 1.13]	.859

Coefficients in bold highlight *p* < .05. Model also controls for which state participants lived in.

## 4. Discussion

The Mental Disorder Prevalence Study (MDPS) provided a unique opportunity to evaluate the impact of type of insurance on the mental health treatment gap in the US between October 2020-October 2022. Our study found that outpatient mental health service utilization differed depending on the type of insurance participants had. Consistent with previous research, treatment rates were lowest among participants who lacked health insurance [9–11]. Among individuals meeting 12-month diagnostic criteria for an MDPS diagnosis, only 27.9% of those with no health insurance received any outpatient mental health treatment in the previous year. Compared to participants with private insurance, individuals with no insurance were significantly less likely to have received any outpatient mental health treatment in the past year, even after adjusting for demographic characteristics and severe functional impairment. Participants in this sample with Medicare, though, had four times greater odds of receiving outpatient mental health services in the past year, compared to participants who had private insurance. Medicare is a federally funded health insurance for people aged 65 and older and for people younger than 65 who have a disability (a category which includes disabling mental disorders). The current study included a non-elderly sample, so all Medicare enrollees had a disability, and our findings suggest that individuals with the greatest need (most complex illness) suffered less disruption in care early in the COVID pandemic [29]. Indeed, Medicare enrollees in this sample had evidence of more severe functional impairment: 42.8% had GAF ≤ 50. Many of these non-elderly Medicare enrollees may have been receiving disability due to a chronic medical condition, and for these individuals, more frequent contacts with the health care system might have provided greater opportunities to access mental health care.

Medicaid enrollees were neither more nor less likely than those with private insurance to have accessed mental health treatment in the past year, after adjusting for demographic characteristics (including income and state of residence). This finding is consistent with previous reports that among low-income adults with mental disorders, Medicaid expansion has been associated with an increased probability of receiving mental health treatment [30], and significant reductions in delaying care or medications for financial reasons [31]. Moreover, the Medicaid continuous coverage provision during the pandemic (i.e., the extension of Medicaid during the public health emergency) may have contributed to more Medicaid enrollees accessing mental health services during the study period (compared to the pre-pandemic period when many Medicaid enrollees experienced periods of going on and off Medicaid). That Medicaid enrollees, unlike Medicare enrollees, were not more likely to access mental health services than participants with private insurance may reflect the persistence of entrenched barriers experienced to a greater extent by Medicaid enrollees such as limited covered services [32], lower reimbursement rates (relative to Medicare) [18] and shortage of psychiatric providers who accept Medicaid [33].

Among individuals with at least one outpatient mental health visit in the previous year, Medicaid enrollees had on average twice as many visits as those with private insurance. This may be explained by the need for more intensive services based on more serious and disabling mental disorders. Consistent with previous national survey studies [30,34], the prevalence of mental disorders in the MDPS was highest among participants who had public insurance. Recent studies have also suggested that an increase in mental health service use following Medicaid expansion was due to an increase in the number of visits among Medicaid enrollees who used services, not an increase in the number of people using services [35]. A simple count of the number of visits should be interpreted with caution, though, as it provides little information about the quality or the appropriateness of the care received.

The COVID pandemic progressed during the MDPS study period, and the moderating effect of time on the relationship between type of insurance and odds of mental health service utilization is interesting. The effect differences between private insurance and Medicare, Medicaid and other insurance were all reduced as the pandemic progressed. Previous studies of mental health services utilization suggested that early in the pandemic, mental health treatment seeking decreased, mainly due to delays in accessing care [36], but this was followed by a return to baseline and then an increase as services pivoted to telehealth [29]. In our study, this pattern of utilization was only observed for participants with private insurance. These findings suggest that those with greatest need (most complex illness) suffered less disruption in care early in the pandemic; but later in pandemic, there was both markedly increased need for services (including among those not previously seeking services) and an increased willingness of some who had dropped out of care because of pandemic issues to return to care.

This study has several key public health implications. First, having any health insurance is associated with greater likelihood of accessing mental health care. Despite the implementation of the Affordable Care Act (ACA) in 2010, there are still 28 million Americans who lack health insurance [37]. While non-financial reasons, such as attitudes about mental illness and seeking care, might contribute to this association, there is substantial evidence that it represents structural barriers to care [10,11]. Second, the mental health treatment gap that was reported more than 20 years ago appears to have persisted despite significant policy changes aimed at increasing access to care and parity of coverage for mental health services. Lack of insurance is just one barrier to mental health care. People with insurance struggle to access mental health services in the US because of shortages of mental health specialists, inaccurate provider directories [16], and expensive treatments and insurance denials [38]. Third, well established racial and ethnic disparities in access to mental health care [39] were also evident in the MDPS sample: participants of Black or Asian race or Hispanic ethnicity were significantly less likely to receive any outpatient mental health treatment in the previous year. Additional barriers exacerbate the treatment gap for these under-served groups, including lack of a diverse mental health care workforce [40], the absence of culturally informed treatment options [41], and stereotypes and discrimination associated with poor mental health [42,43].

The study has several important strengths that increase the importance of the findings. Diagnoses of mental disorders were based on semi-structured psychiatric interviews conducted by trained clinicians, and the presence of a clinical diagnosis increases the confidence that participants needed mental health treatment. Second, the survey was based on a national household probability sample and included a large number of participants. Third, the inclusion of a clinician assessment of severity of functional impairment provided the opportunity to examine the association between severity of mental disorder and utilization of outpatient treatment.

Study findings need to be interpreted in the context of several limitations. First, utilization of services was determined based on response to only a few questions that do not capture nuances related to accessing care. Lack of service use might reflect either barriers to access or attitudinal factors. Moreover, the survey provided limited information about quality and appropriateness of mental health care. Second, the low overall MDPS survey response rate reduces the representativeness of the sample and the generalizability of the results. It is likely that the 3-stage design contributed to the low response rate, particularly because the COVID pandemic disrupted planned in-person rostering and screening data collection. In-person surveys typically have higher response rates [44,45]. The COVID pandemic had substantial impact on response rates of large survey studies [46], and the MDPS response rate was consistent with these effects of the pandemic. Third, access to either the virtual Zoom platform or a telephone was required for participation in the clinical interview and access to technology may have been a barrier for some potential participants. One-third of the interviews were conducted by telephone [22], which may have mitigated this potential bias. Fourth, many individuals who did not meet criteria for an MDPS disorder may have needed treatment for a different mental disorder (e.g., panic disorder, borderline personality disorder), but prevalence of and utilization of services for these disorders were not addressed in the MDPS study [47]. Finally, the survey did not provide detailed information about insurance (e.g., covered mental health services); heterogeneity across private insurance types in particular makes it difficult to draw conclusions about the success of the large private insurance marketplace in meeting the mental health care needs of the privately-insured population. In particular, Medicare enrollees were not asked whether they were enrolled in a Medicare Advantage plan (i.e., a Medicare-approved plan from a private insurance company). Categorizing these participants with other Medicare enrollees, though, would under-estimate the differences between participants with private insurance and those with Medicare.

## 5. Conclusion

The MDPS survey, conducted between 2020–2022 and the first national study to use DSM-5 based semi-structured clinical interviews within a large-scale population survey, provides evidence of persisting mental health treatment gaps. The study found that people without health insurance continue to be the least likely to access mental health care, regardless of diagnosis or severity of illness. Even among individuals with health insurance, though, there are substantial disparities in access to mental health care, particularly among people of color. Throughout the study, Medicare enrollees, who had on average the greatest severity of illness and functional impairment, had much greater odds of mental health service utilization. Over the course of the pandemic, an increased likelihood of accessing mental health services was seen only among participants with private insurance. Low levels of mental health care among adults with mental disorders suggest the need for policy efforts to address systemic barriers to mental health care.
